# Supplementary material for: A Likelihood Approach for Real-Time Calibration of Stochastic Compartmental Epidemic Models
Source: PLoS Comput Biol. 2017 Jan 17;13(1):e1005257. doi: 10.1371/journal.pcbi.1005257 (PMC5240920; doi:10.1371/journal.pcbi.1005257)
Supplement: S1 File — (TAR.GZ) [file pcbi.1005257.s014.tar.gz › HSPH_Online-SI-Revision/MSS10/n1-mild/n1-mild_NEW_table8.pdf]

1	R0	{1.27677, 7.89375, 1.}	{1.25552, 5.11221, 1.53}	{{1.24271, 3.98513, 0.}, {1.28596, 5.50828, 2.}}	{2.08659, 35.2372, 99.}	inf	4.	7.05133	{2.65827, 12.2474}	91.188	0.269368	newly	2.	8.79	{3., 16.}	345.5	0.594134	Reff	0.856713	0.83865	{0.821277, 0.864485}	2.48543	ar	400.	365.54	{329., 408.}	11.255
2	R0	{1.33231, 10.5259, 2.}	{1.32774, 3.92617, 0.2}	{{1.25474, 3.26445, 0.}, {1.44762, 4.25564, 1.}}	{5.23298, 62.7, 90.}	inf	6.	2.7413	{1.29039, 4.05509}	55.6717	0.393374	newly	4.	4.11	{1., 8.}	55.75	0.287258	Reff	0.714118	0.71596	{0.676052, 0.781746}	4.97632	ar	467.	468.86	{459., 485.}	1.91435
3	R0	{1.2273, 6.76944, 1.}	{1.2367, 2.88348, 0.}	{{1.22544, 2.5364, 0.}, {1.25474, 3.26445, 0.}}	{1.8935, 57.4044, 100.}	inf	9.	10.2921	{2.45529, 19.7264}	56.2481	0.252532	newly	6.	20.3	{4., 41.}	249.	0.482223	Reff	0.857883	0.862814	{0.85089, 0.879871}	1.91969	ar	314.	345.89	{300., 410.}	12.4045
4	R0	{1.41215, 17.5742, 3.}	{1.13682, 3.76228, 0.28}	{{1.06738, 2.19254, 0.}, {1.25474, 6.0871, 1.}}	{19.4971, 78.592, 90.6667}	inf	25.	7.2729	{2.12656, 13.8162}	70.9084	0.630451	newly	8.	13.29	{3., 26.}	100.625	0.309409	Reff	1.09159	0.89885	{0.841865, 0.979916}	17.657	ar	465.	257.	{206., 349.}	44.8387
5	R0	{1.42222, 6.15886, 0.1}	{1.41901, 3.17623, 0.14}	{{1.24751, 1.89883, 0.}, {1.47608, 4.06661, 1.}}	{3.88119, 48.4283, 214.}	inf	11.	4.62709	{2.05823, 7.77482}	58.1564	0.42811	newly	5.	8.23	{3., 15.}	87.	0.268685	Reff	0.742399	0.749734	{0.662254, 0.782091}	4.22253	ar	490.	485.25	{470., 506.}	2.76122
6	R0	{1.24363, 9.95596, 0.1}	{1.25463, 3.50185, 0.08}	{{1.22544, 3.07157, 0.}, {1.28596, 3.98513, 0.}}	{2.11623, 64.8266, 164.}	inf	11.	10.9944	{4.12327, 19.3816}	45.0809	0.22384	newly	12.	19.24	{6., 35.}	84.5	0.275995	Reff	0.863079	0.870739	{0.842114, 0.899069}	2.22904	ar	411.	355.33	{305., 416.}	15.6375
7	R0	{1.36478, 5.96056, 1.}	{1.39193, 1.893, 0.01}	{{1.39162, 1.89883, 0.}, {1.39162, 1.89883, 0.}}	{1.98925, 68.2412, 99.}	inf	8.	10.0352	{2.11809, 19.336}	65.6696	0.274036	newly	5.	24.19	{4., 47.}	394.6	0.608108	Reff	0.754723	0.766902	{0.752462, 0.77774}	1.81777	ar	484.	474.49	{443., 510.}	5.10124
8	R0	{1.36335, 17.6429, 2.}	{1.1767, 5.87404, 1.23}	{{1.17082, 5.71232, 1.}, {1.19306, 6.16288, 2.}}	{13.6908, 66.7059, 40.5}	inf	28.	12.1636	{4.2572, 19.4383}	56.5586	0.41759	newly	12.	13.82	{5., 23.}	45.	0.199643	Reff	1.02251	0.901123	{0.883251, 0.922272}	11.8717	ar	484.	290.25	{239., 389.}	40.031
9	R0	{1.26575, 5.28707, 0.1}	{1.45835, 4.02846, 0.04}	{{1.44762, 3.83841, 0.}, {1.47608, 4.06661, 0.}}	{15.2166, 23.8054, 136.}	inf	6.	5.87613	{1.77009, 10.3476}	49.9907	0.250659	newly	5.	8.81	{2., 16.}	107.	0.334138	Reff	0.669582	0.77164	{0.761248, 0.784975}	15.2421	ar	472.	488.88	{467., 515.}	3.98305
10	R0	{1.42942, 9.49905, 2.}	{1.41515, 8.5101, 1.2}	{{1.36632, 6.77341, 1.}, {1.42801, 9.02643, 2.}}	{0.998346, 10.411, 40.}	inf	1.	6.21422	{2.674, 10.8655}	521.422	0.737451	newly	1.	4.59	{2., 8.}	363.	0.609061	Reff	0.760451	0.745449	{0.723741, 0.756186}	1.97279	ar	468.	490.75	{471., 521.}	4.86111
11	R0	{1.46827, 15.0983, 0.1}	{1.16411, 5.11731, 0.32}	{{1.10202, 3.98513, 0.}, {1.28596, 5.77111, 1.}}	{20.7157, 66.1067, 370.}	inf	22.	7.35784	{2.65148, 12.7937}	66.5553	0.545996	newly	11.	9.71	{3., 20.}	46.8182	0.276874	Reff	1.12469	0.908707	{0.860709, 1.0049}	19.2041	ar	495.	261.35	{218., 338.}	47.202
12	R0	{1.25751, 13.279, 1.}	{1.10464, 2.55867, 0.05}	{{1.06738, 2.19254, 0.}, {1.22544, 3.26445, 0.}}	{12.2474, 80.7314, 95.}	inf	16.	5.70708	{1.676, 10.6714}	64.7104	0.537938	newly	9.	14.14	{3., 32.}	96.6667	0.319886	Reff	0.975828	0.868528	{0.836156, 0.967468}	11.2655	ar	366.	250.49	{211., 333.}	32.6366
13	R0	{1.3425, 9.16361, 1.}	{1.35275, 6.03459, 1.81}	{{1.34436, 4.98002, 1.}, {1.3618, 6.25107, 2.}}	{0.763299, 34.1461, 91.}	inf	2.	3.74183	{1.64511, 6.22294}	94.812	0.264809	newly	0.1	4.02	{1., 7.}	3926.	1.48791	Reff	0.753143	0.756538	{0.748653, 0.762323}	0.836817	ar	442.	452.59	{439., 473.}	2.63575
14	R0	{1.18936, 6.20583, 0.1}	{1.28102, 4.82466, 1.2}	{{1.27231, 3.98513, 0.}, {1.28596, 5.40945, 2.}}	{7.70665, 22.256, 1176.}	inf	3.	4.53742	{1.53281, 8.20047}	71.883	0.230575	newly	3.	6.01	{2., 11.}	118.333	0.344641	Reff	0.735024	0.789715	{0.774498, 0.796868}	7.44061	ar	382.	395.9	{382., 416.}	3.68063
15	R0	{1.27137, 9.78868, 0.1}	{1.30486, 4.20245, 0.4}	{{1.28596, 3.98513, 0.}, {1.3618, 4.98002, 1.}}	{2.66001, 57.0683, 440.}	inf	16.	9.23367	{2.84889, 15.7863}	47.4435	0.338135	newly	13.	12.93	{4., 25.}	51.9231	0.267382	Reff	0.752651	0.781284	{0.763749, 0.819611}	3.80433	ar	496.	426.56	{398., 458.}	14.5403
16	R0	{1.23102, 13.6452, 1.}	{1.27733, 5.25007, 1.76}	{{1.21937, 4.98002, 1.}, {1.3618, 6.0993, 2.}}	{4.21638, 61.5244, 92.}	inf	26.	11.6337	{3.74958, 17.8547}	56.5063	0.425722	newly	13.	16.42	{4., 22.}	60.9231	0.227594	Reff	0.853097	0.903472	{0.861098, 0.967175}	5.90498	ar	445.	346.29	{293., 412.}	22.5685
17	R0	{1.27213, 13.7452, 3.}	{1.15514, 5.82501, 0.63}	{{1.13227, 5.71232, 0.}, {1.18976, 6.16288, 1.}}	{9.19666, 57.6215, 79.}	inf	30.	9.80306	{2.69507, 17.7572}	67.3231	0.564244	newly	16.	11.03	{3., 20.}	43.4375	0.310917	Reff	0.957914	0.893134	{0.872103, 0.914739}	6.76262	ar	367.	282.45	{225., 365.}	24.5422
18	R0	{1.25318, 11.2151, 0.1}	{1.27424, 5.52398, 1.88}	{{1.24271, 4.98002, 1.}, {1.29927, 6.78013, 2.}}	{1.99768, 50.7451, 1784.}	inf	24.	9.80748	{4.32831, 15.9758}	59.1355	0.441911	newly	17.	11.49	{4., 20.}	41.9412	0.283715	Reff	0.833365	0.86541	{0.843493, 0.874073}	3.84535	ar	416.	359.86	{322., 416.}	14.9663
19	R0	{1.46006, 8.11496, 0.1}	{1.44761, 4.02337, 0.05}	{{1.44762, 3.83841, 0.}, {1.47488, 4.06661, 0.}}	{1.53917, 50.4203, 144.}	inf	20.	7.76373	{2.14096, 13.1841}	61.1814	0.494461	newly	12.	11.51	{3., 21.}	48.4167	0.274464	Reff	0.762151	0.773348	{0.76125, 0.793424}	2.52157	ar	496.	488.43	{463., 523.}	4.37298
20	R0	{1.14273, 6.71261, 0.1}	{1.26794, 3.7515, 0.11}	{{1.21159, 3.26445, 0.}, {1.28596, 3.98513, 1.}}	{10.9573, 44.1126, 188.}	inf	15.	5.54222	{2.2649, 9.92942}	63.3988	0.496697	newly	11.	8.8	{2., 15.}	42.7273	0.262184	Reff	0.742775	0.836158	{0.801439, 0.851748}	12.5722	ar	362.	363.71	{339., 399.}	5.45028
21	R0	{1.42432, 8.23905, 1.}	{1.24597, 2.89144, 0.}	{{1.22544, 2.5364, 0.}, {1.25474, 3.26445, 0.}}	{12.5219, 64.9056, 100.}	inf	6.	5.76526	{1.46552, 10.5641}	48.9076	0.253303	newly	5.	10.63	{2., 22.}	135.	0.353197	Reff	0.853168	0.746641	{0.733057, 0.756066}	12.4861	ar	405.	417.23	{397., 449.}	4.03704
22	R0	{1.29386, 17.863, 2.}	{1.27988, 9.68345, 1.93}	{{1.19093, 6.83219, 2.}, {1.29096, 10.0371, 2.}}	{1.0802, 45.7905, 5.5}	inf	35.	31.5197	{20.1341, 41.1814}	20.3666	0.112175	newly	16.	22.12	{13., 30.}	47.625	0.174268	Reff	0.98204	0.975688	{0.935588, 0.99134}	1.20592	ar	488.	402.64	{298., 490.}	18.0656
23	R0	{1.39274, 13.0167, 5.}	{1.44604, 3.9459, 0.}	{{1.44762, 3.83841, 0.}, {1.47608, 4.06661, 0.}}	{4.9129, 69.6859, 100.}	inf	19.	10.7951	{2.7554, 18.8575}	44.9059	0.337711	newly	8.	16.91	{4., 30.}	132.625	0.367486	Reff	0.799433	0.841893	{0.827556, 0.86307}	6.23605	ar	475.	457.58	{413., 522.}	8.53895
24	R0	{1.32178, 15.8991, 1.}	{1.3063, 6.24561, 2.74}	{{1.27231, 5.40945, 2.}, {1.31223, 6.58803, 3.}}	{1.30022, 60.7172, 188.}	inf	28.	13.8383	{4.99804, 21.0128}	50.8613	0.37271	newly	13.	13.52	{3., 21.}	39.6923	0.20584	Reff	0.810251	0.819219	{0.805872, 0.833169}	1.4313	ar	471.	422.85	{369., 481.}	11.8153
25	R0	{1.37568, 17.8427, 1.}	{1.17751, 5.86596, 1.26}	{{1.17082, 5.77111, 1.}, {1.19018, 6.16288, 2.}}	{14.4054, 67.124, 32.}	inf	24.	7.35965	{2.76089, 12.0656}	69.3348	0.576492	newly	8.	8.11	{3., 13.}	43.375	0.220983	Reff	1.00837	0.882695	{0.872604, 0.895539}	12.4635	ar	398.	284.62	{249., 341.}	28.4975
26	R0	{1.20993, 6.4656, 0.1}	{1.23195, 5.8133, 1.94}	{{1.21937, 5.50828, 2.}, {1.24271, 6.0993, 2.}}	{1.82009, 10.4779, 1846.}	inf	2.	6.02588	{2.52099, 10.8134}	204.306	0.429661	newly	1.	6.5	{2., 12.}	552.	0.723732	Reff	0.803394	0.813058	{0.801015, 0.823956}	1.32938	ar	345.	367.52	{338., 419.}	7.37391
27	R0	{1.20548, 13.8003, 0.1}	{1.25254, 3.5272, 0.11}	{{1.22544, 3.07157, 0.}, {1.28596, 3.98513, 0.}}	{4.13313, 74.4411, 194.}	inf	23.	9.21465	{1.82278, 16.2497}	61.0036	0.509781	newly	10.	15.62	{3., 29.}	90.4	0.314299	Reff	0.817315	0.866456	{0.8441, 0.894354}	6.20407	ar	350.	345.26	{303., 410.}	10.1771
28	R0	{1.3176, 14.6594, 1.}	{1.21775, 4.15761, 0.37}	{{1.10202, 3.57392, 0.}, {1.28596, 5.30671, 2.}}	{7.57841, 71.6386, 89.}	inf	16.	8.54973	{2.81356, 15.2005}	48.1635	0.353558	newly	8.	14.85	{3., 28.}	111.125	0.337175	Reff	1.06989	0.997774	{0.904013, 1.05842}	6.74932	ar	362.	269.42	{179., 397.}	30.8785
29	R0	{1.28202, 6.23047, 1.}	{1.28488, 3.98149, 0.16}	{{1.25474, 3.26445, 0.}, {1.28596, 3.98513, 0.}}	{0.820753, 36.2113, 98.}	inf	4.	6.90015	{2.48952, 11.7294}	87.4071	0.259031	newly	5.	9.9	{3., 18.}	116.4	0.322435	Reff	0.782032	0.780045	{0.765397, 0.787068}	0.992063	ar	391.	410.98	{390., 436.}	5.22251
30	R0	{1.28177, 8.83252, 1.}	{1.31967, 5.76556, 2.04}	{{1.28596, 3.98513, 0.}, {1.3618, 6.58803, 3.}}	{3.01374, 34.7235, 134.}	inf	18.	11.411	{4.22262, 18.3338}	39.8629	0.274453	newly	11.	12.07	{4., 20.}	44.8182	0.217378	Reff	0.76778	0.799201	{0.780576, 0.830479}	4.12704	ar	427.	430.76	{392., 481.}	6.49649
31	R0	{1.35886, 5.97708, 0.1}	{1.39515, 6.44427, 0.09}	{{1.38815, 5.7404, 0.}, {1.40191, 6.71734, 0.}}	{2.67065, 13.7163, 176.}	inf	0.1	5.23375	{2.44193, 7.91168}	5133.75	1.66876	newly	0.1	5.2	{2., 9.}	5100.	1.64183	Reff	0.69166	0.702837	{0.69564, 0.706534}	1.62389	ar	491.	508.66	{494., 528.}	3.59674
32	R0	{1.27601, 17.9804, 3.}	{1.21295, 3.54584, 0.17}	{{1.06738, 2.19254, 0.}, {1.28596, 4.21286, 1.}}	{5.90748, 80.2794, 94.3333}	inf	36.	11.3459	{3.51538, 20.7296}	68.4836	0.58036	newly	15.	22.03	{6., 43.}	75.	0.262292	Reff	0.962112	0.944418	{0.831431, 1.00711}	6.02739	ar	445.	299.26	{221., 423.}	33.7573
33	R0	{1.3368, 11.3235, 0.1}	{1.3289, 7.46425, 2.95}	{{1.31223, 6.58803, 3.}, {1.34668, 8.42466, 3.}}	{1.35252, 34.0818, 2850.}	inf	13.	12.468	{6.39386, 18.3452}	28.6565	0.139465	newly	7.	10.29	{5., 15.}	57.5714	0.190044	Reff	0.788712	0.784717	{0.771471, 0.79698}	1.09554	ar	417.	447.56	{413., 483.}	7.80815
34	R0	{1.21021, 11.3635, 2.}	{1.17416, 4.75992, 0.66}	{{1.08063, 2.5364, 0.}, {1.24271, 5.77111, 1.}}	{4.25309, 58.1122, 67.}	inf	18.	7.42031	{2.81785, 12.6402}	58.7761	0.444468	newly	15.	10.91	{3., 20.}	45.1333	0.301931	Reff	0.905237	0.890724	{0.815732, 0.936341}	3.77013	ar	327.	282.83	{242., 347.}	17.5872
35	R0	{1.53382, 10.8866, 0.1}	{1.39886, 8.29335, 0.04}	{{1.39882, 8.26611, 0.}, {1.39882, 8.26611, 0.}}	{8.7988, 24.0939, 136.}	inf	21.	29.5246	{21.9951, 36.5482}	43.4145	0.150871	newly	15.	22.37	{15., 29.}	52.2	0.174843	Reff	0.852804	0.76582	{0.755847, 0.776072}	10.1997	ar	476.	520.19	{477., 560.}	9.65336
36	R0	{1.20423, 6.83844, 0.1}	{1.28362, 3.87475, 0.02}	{{1.25474, 3.26445, 0.}, {1.28596, 3.98513, 0.}}	{6.59248, 43.3387, 116.}	inf	0.1	3.26058	{1.54998, 4.98075}	3160.58	1.46871	newly	0.1	5.15	{2., 10.}	5050.	1.62323	Reff	0.70327	0.745445	{0.728787, 0.749007}	5.99693	ar	416.	429.31	{419., 452.}	3.19952
37	R0	{1.43629, 10.1548, 3.}	{1.28742, 4.03019, 0.17}	{{1.28596, 3.98513, 0.}, {1.28596, 3.98513, 0.}}	{10.4187, 60.3125, 94.3333}	inf	12.	7.47737	{2.00601, 14.1392}	45.7348	0.31632	newly	8.	11.82	{3., 23.}	76.75	0.25645	Reff	0.963751	0.869688	{0.859109, 0.876227}	9.83473	ar	367.	357.85	{321., 412.}	9.0109
38	R0	{1.37504, 8.93428, 1.}	{1.45818, 3.93497, 0.}	{{1.44762, 3.83841, 0.}, {1.47608, 4.06661, 0.}}	{6.41719, 55.9565, 100.}	inf	7.	6.2008	{1.97274, 9.90371}	36.8062	0.210862	newly	5.	9.38	{3., 16.}	107.6	0.316351	Reff	0.717771	0.762329	{0.752361, 0.774883}	6.56729	ar	483.	495.17	{474., 531.}	3.46377
39	R0	{1.51637, 9.36742, 1.}	{1.3351, 4.6612, 0.87}	{{1.28596, 3.98513, 0.}, {1.3618, 4.98002, 2.}}	{11.9539, 50.2403, 47.}	inf	9.	6.64363	{2.37713, 10.986}	37.4108	0.233619	newly	4.	8.54	{2., 15.}	130.5	0.354298	Reff	0.884044	0.78149	{0.752386, 0.801445}	11.6005	ar	441.	435.55	{411., 470.}	5.13152
40	R0	{1.23658, 9.82869, 1.}	{1.27715, 3.70897, 0.03}	{{1.22544, 3.07157, 0.}, {1.28596, 3.98513, 0.}}	{3.50148, 62.2639, 99.}	inf	1.	5.0312	{1.57563, 9.35541}	403.12	0.621179	newly	1.	7.95	{2., 16.}	695.	0.779965	Reff	0.724636	0.743249	{0.715706, 0.752832}	2.92133	ar	414.	433.13	{416., 465.}	4.62077
41	R0	{1.24247, 5.4242, 0.1}	{1.26164, 2.58646, 0.06}	{{1.24751, 1.89883, 0.}, {1.31748, 2.5364, 0.}}	{1.85238, 52.3473, 154.}	inf	5.	5.61951	{1.91285, 9.01144}	49.496	0.212407	newly	5.	13.24	{3., 25.}	184.	0.400061	Reff	0.821273	0.833147	{0.819602, 0.872765}	1.87184	ar	345.	360.18	{337., 399.}	5.6
42	R0	{1.28208, 4.40223, 0.1}	{1.23907, 2.53364, 0.}	{{1.24751, 2.5364, 0.}, {1.24751, 2.5364, 0.}}	{3.69654, 42.4465, 100.}	inf	0.1	4.29905	{1.55738, 7.21925}	4199.05	1.57691	newly	0.1	10.05	{3., 18.}	9950.	1.89957	Reff	0.847455	0.81366	{0.812839, 0.822663}	4.25489	ar	339.	360.76	{342., 399.}	6.41888
43	R0	{1.56773, 6.50474, 0.1}	{1.42499, 3.92715, 0.08}	{{1.28596, 3.83841, 0.}, {1.47608, 4.06661, 0.}}	{9.1047, 39.6264, 174.}	inf	3.	2.70814	{1.16961, 4.53142}	36.5023	0.185735	newly	2.	3.71	{1., 7.}	100.5	0.280134	Reff	0.790136	0.718612	{0.647222, 0.745727}	9.05212	ar	498.	501.89	{495., 512.}	1.20281
44	R0	{1.10489, 6.01941, 1.}	{1.23473, 3.11132, 0.03}	{{1.22544, 3.07157, 0.}, {1.25474, 3.26445, 0.}}	{11.8192, 48.3119, 97.}	inf	1.	2.91536	{1.28628, 4.95921}	191.536	0.40893	newly	0.1	5.63	{1., 12.}	5530.	1.62228	Reff	0.724808	0.807615	{0.798134, 0.821819}	11.4938	ar	346.	355.17	{345., 371.}	2.81792
45	R0	{1.25888, 14.1926, 1.}	{1.36399, 4.93822, 1.01}	{{1.3618, 4.94504, 1.}, {1.3618, 4.98002, 1.}}	{8.34948, 65.2057, 11.}	inf	17.	6.16479	{1.81842, 10.7093}	63.8357	0.51179	newly	7.	7.61	{2., 14.}	51.8571	0.239674	Reff	0.682313	0.754062	{0.745747, 0.759529}	10.5155	ar	471.	463.02	{445., 483.}	3.64331
46	R0	{1.45292, 16.616, 2.}	{1.20891, 5.81288, 1.67}	{{1.17082, 5.50828, 1.}, {1.24271, 6.0993, 2.}}	{16.7947, 65.0164, 16.5}	inf	28.	10.9927	{4.27684, 18.5217}	60.7403	0.467132	newly	9.	12.12	{4., 21.}	58.8889	0.221509	Reff	1.03012	0.877653	{0.848854, 0.907894}	14.8009	ar	494.	326.41	{273., 390.}	33.9251
47	R0	{1.22528, 16.6033, 0.1}	{1.14157, 6.14525, 0.28}	{{1.13227, 5.71232, 0.}, {1.17082, 6.0871, 1.}}	{6.8318, 63.0423, 346.}	inf	30.	10.163	{3.82913, 16.5165}	66.1235	0.535778	newly	10.	11.53	{4., 19.}	48.3	0.215037	Reff	1.00963	0.963281	{0.95202, 0.986631}	4.59074	ar	368.	218.54	{158., 300.}	41.0054
48	R0	{1.1669, 6.92715, 0.1}	{1.38586, 1.92433, 0.}	{{1.39162, 1.89883, 0.}, {1.39162, 1.89883, 0.}}	{18.7642, 72.2204, 100.}	inf	6.	6.80899	{1.62177, 13.123}	56.8622	0.255685	newly	3.	20.67	{3., 44.}	597.	0.717057	Reff	0.735147	0.871971	{0.8629, 0.882809}	18.6117	ar	416.	398.78	{367., 446.}	7.55288
49	R0	{1.32749, 16.0887, 3.}	{1.13477, 5.89553, 0.18}	{{1.10202, 5.30671, 0.}, {1.17082, 6.0871, 1.}}	{14.5177, 63.4691, 95.3333}	inf	18.	9.05108	{3.75846, 14.3891}	51.1087	0.364825	newly	3.	10.57	{4., 20.}	258.333	0.500307	Reff	1.06863	0.923616	{0.90283, 0.941268}	13.5701	ar	353.	234.23	{185., 312.}	34.4108
50	R0	{1.29636, 15.2609, 0.1}	{1.30881, 4.19086, 0.29}	{{1.28596, 3.98513, 0.}, {1.3618, 4.98002, 1.}}	{2.19579, 72.5386, 340.}	inf	33.	9.43658	{3.52472, 14.4362}	71.4043	0.61114	newly	13.	13.35	{4., 21.}	45.9231	0.23242	Reff	0.735036	0.772885	{0.752745, 0.806615}	5.14921	ar	470.	439.05	{408., 487.}	8.75957
